# The chitosan/tri-calcium phosphate bio-composite bone cement promotes better osteo-integration: an in vitro and in vivo study

**DOI:** 10.1186/s13018-019-1201-2

**Published:** 2019-05-29

**Authors:** Chih-Hsiang Fang, Yi-Wen Lin, Jui-Sheng Sun, Feng-Huei Lin

**Affiliations:** 1 Institute of Biomedical Engineering, College of Medicine and College of Engineering, National Taiwan University, No. 1, Sec. 4, Roosevelt Rd, Taipei, 10617 Taiwan; 2 Department of Orthopedic Surgery, National Taiwan University Hospital, No. 7, Chung-Shan South Road, Taipei, 10002 Taiwan; 3 Division of Biomedical Engineering and Nanomedicine Research, National Health Research Institutes, No. 35, Keyan Road, Zhunan, Miaoli County, 35053 Taiwan; 4 Department of Orthopedic Surgery, College of Medicine, National Taiwan University, No. 1, Sec. 1, Ren-Ai Rd, Taipei, 10051 Taiwan

**Keywords:** Tri-calcium phosphate (TCP), Chitosan, Polymethylmethacrylate (PMMA), Bio-composite bone cement

## Abstract

**Background:**

Polymethylmethacrylate bone cement has a variety of applications in orthopedic surgery, but it also has some shortcomings such as high heat generation during polymerization and poor integration with bone tissue. In this study, a bio-composite bone cement composed of tri-calcium phosphate and chitosan as additives to acrylic bone cement was developed. Our hypothesis is that this new bio-composite bone cement has a better osteo-integration than pure polymethyl methacrylate cement.

**Methods:**

Physiological composition, i.e., 65 wt% inorganic and 35 wt% organic components, of tri-calcium phosphate and chitosan contents was selected as degradable additives to replace acrylic bone cement. A series of properties such as exothermic temperature changes, setting time, bio-mechanical characteristics, degradation behaviors, and in vitro cytotoxicity were examined. Preliminary in vivo animal study was also performed.

**Results:**

The results showed that the bio-composite bone cement exhibited lower curing temperature, longer setting time, higher weight loss and porosity after degradation, lower compressive Young’s modulus, and ultimate compressive strength as compared with those of pure polymethyl methacrylate cement. Cell proliferation tests demonstrated that the bio-composite bone cement was non-cytotoxic, and the in vivo tests revealed that was more osteo-conductive.

**Conclusions:**

The results indicated that the modified chitosan/tri-calcium phosphate/polymethyl methacrylate bio-composites bone cement could be degraded gradually and create rougher surfaces that would be beneficial to cell adherence and growth. This new bio-composite bone cement has potential in clinical application. Our future studies will focus on long-term implantation to investigate the stability of the bio-composite bone cement in long-term implantation.

## Introduction

Polymethylmethacrylate (PMMA) bone cement has a variety of applications in orthopedic surgery. Because of its excellent mechanical properties, suitable color, and injectable application, acrylic bone cement has been used in orthopedic surgeries such as vertebroplasty, prosthetic replacement for joints, or bone filler after severe fracture with bone defect [1–3]. Primary uses of PMMA in total arthroplasties are limited to prostheses fixation and antibiotic delivery. Current joint placement surgeries have shown over 90% success over the last 10 years, with failure mainly caused by aseptic loosening and tissue necrosis [4–6]. Aseptic loosening as a result of wear debris is considered to be the main cause of long-term implant failure in orthopedic surgery and improved biomaterials for bearing surfaces decreases significantly the release of micrometric wear particles [7]. With the large number of total joint arthroplasties expected to continue to rise, understanding the role bone cement plays in the success of total joint arthroplasty can have a significant impact on daily practice [8].

However, there are inherent problems in acrylic bone cement. First, the high exothermic reaction temperature during its polymerization (hardening) process has been shown to damage the surrounding tissues. Second, acrylic bone cement has poor compatibility with the surrounding bone tissue because it neither adheres to the bone nor induces bone formation, thus contributing to its long-term loosening [9, 10]. Several researchers improved the biological characteristics of acrylic bone cement by adding inorganic salts into the matrix of acrylic bone cement, such as tri-calcium phosphate (TCP) or hydroxyapatite (HA). Researchers have found that the addition of inorganic salts can enhance the mechanical properties and bio-compatibility of acrylic bone cement [11–13]; the inorganic salts were also found to reduce the heat generated during hardening [14, 15].

HA is a bio-resorbable calcium phosphate, which shows excellent bio-compatibility with bone tissue; however, the resorption rate of HA is too slow in the physiological environment and thus results in an undesirable long-term HA ceramic inter-surface effect in the implant [13]. TCP is another bio-resorbable and bio-compatible ceramic material with similar characteristics as that of the inorganic phase of natural bone. Clinical practice and experience has shown that TCP has a more favorable resorption pattern and osteo-transduction property than HA does. Besides, TCP is also gradually absorbed following new bone formation and is often incorporated as an additive in bone grafts and dental materials [16–18]. Another potential additive is chitosan. Owing to its characteristics such as bio-compatibility, biodegradability, osteoinduction, and nontoxicity, chitosan is commonly used in orthopedic applications to provide mechanical support for bone regeneration [19, 20]. Furthermore, because of its intramolecular hydrogen bonds, chitosan has a high resistance to heat [21]. Therefore, incorporation of chitosan as an additive is beneficial compared with other organic materials such as collagen, gelatin, and alginate.

The aim of this study was to prepare a bio-composite bone cement with TCP and chitosan as additives to pure acrylic bone cement. TCP and chitosan contents were selected to mimic the composition of human bone, i.e., 65 wt% inorganic and 35 wt% organic components. We examined a series of properties such as exothermic temperatures, setting time, ultimate compressive strength, modulus of compression, degradation behaviors, and shear stress as well as cytotoxicity via in vitro tests. Moreover, preliminary in vivo animal studies were also performed. Our hypothesis is that this new bio-composite bone cement has a better osteo-integration than pure PMMA cement.

## Materials and methods

### Preparation of bio-composite bone cement

Tri-calcium phosphate (TCP; Sigma-Aldrich, St. Louis, MO, USA) and chitosan (Sigma-Aldrich, St. Louis, MO, USA) contents were selected according to the composition of human bones, i.e., an inorganic to organic ratio of 65:35 (*w*/*w*), to act as an additives of PMMA (Howmedica Int. Ltd, Ireland). First, TCP and chitosan powders were mixed in the ratio 65:35 by weight. Then, PMMA was added to this mixture in different ratios (PMMA to TCP/chitosan composition ratios of 3:1, 2:1, 1:1, 1:2, and 1:3 (*w*/*w*) by weight) for a fixed total weight. Finally, MMA monomer was added to the above-mentioned mixture of PMMA and TCP/chitosan in the ratio 2:1 (*w*/*w*) and stirred. After 24 h mixing, the mixed dough was poured into a Teflon cylindrical mold (10 mm in diameter and 10 mm in depth) and set. All reactions were performed at room temperature. Six samples were prepared for examination: five types of bio-composite bone cement with PMMA to TCP/chitosan composition ratios of 3:1, 2:1, 1:1, 1:2, and 1:3 (*w*/*w*), and pure PMMA bone cement as the control group (Table 1). The solidified materials were removed from the mold for subsequent tests. A scanning electron microscope (SEM; Hitachi-S3400, Japan) was used to observe the surface morphologies of the specimens.

**Table 1:** Composition of the specimens (wt%)

TCP powder:chitosan powder = 65:35 (*w*/*w*)			
Item	PMMA	TCP	Chitosan
Pure P	100	0	0
P3TC1	75	16.25	8.75
P2TC1	66.6	21.71	11.69
P1TC1	50	32.5	17.5
P1TC2	32.4	43.29	23.31
P1TC3	25	48.75	26.25

### Mechanical properties of bio-composite bone cement

The mechanical properties of the bio-composite bone cements (cylindrical specimens with 25-mm height and 5-mm diameter) were measured by using a mechanical testing system (QTest/10; MTS Systems Corporation, Eden Prairie, MN, USA). All samples were compressed or bent at a speed of 10 mm/min, and all the mechanical parameters were automatically recorded by a computer. The shear stress between the specimens and metal was also estimated.

### In vitro degradation test

The prepared specimens were immersed in a tube containing 15 mL of phosphate buffer saline solution (pH 7.2) and then placed in a shaker at 37 °C for slow shaking. After two months of degradation, the morphological changes in the specimens were observed using a SEM (Hitachi-S3400, Japan). The degradation ratio was expressed as weight loss of the specimens per twenty days, i.e., weight loss (%) = [(Wd − Wi)/Wi] × (− 1) × 100, where Wi is the initial weight of the specimen and Wd is the weight of the dried sample after degradation.

### Solidification of bio-composite bone cement

The exothermic temperatures and intrusion lengths were measured during solidification. The temperature change during solidification was measured using a digital thermometer (Digital thermometer, Model 305, Taiwan), and the setting time was calculated according to ASTM F451- 99A [22]: setting time = (Tamb + Tmax)/2, where Tamb is the ambient temperature (25 °C) and Tmax is the maximum temperature.

### Cytotoxicity

Mouse fibroblast cell line (L929, Thermo Fisher Scientific, Carlsbad, CA, USA) was seeded onto the specimens (height = 10 mm, diameter = 10 mm) at a density of 2 × 10^4^ cells/mL and then cultured with high-glucose Dulbecco’s Modified Eagle’s Medium (DMEM-HG, Sigma-Aldrich, St. Louis, MO, USA) with 10% fetal bovine serum (FBS, Gibco BRL, Cheshire, UK) and 1% penicillin/streptomycin for 72 h. The cell activity was determined by the MTT [3-(4,5-dimethylthiazol-2-yl)-2,5,-diphenyl-tetrazolium bromide] assay. The MTT reagent (5 mg/mL) was added to each well and incubated for 3 h in an incubator at 37 °C. After removing the medium, 1 mL DMSO was added to each well, and the microtiter plate was shaken for 15 min. Then, ODs of the purple solution in the wells were determined at 570 nm with an ELISA plate reader.

### Animal study

Adult eight-week-oldSprague-Dawley (SD) rats, weighing 200–250 g, were used for animal study. Rats were kept in individual cages with free access to specific rat chow and water prior to and after surgery. The cages were placed in a temperature- and humidity-controlled room with 12-h light cycles. The animals were anesthetized by an intramuscular injection of a 1.1 mixture of ketamine (50 mg/mL; ketamine hydrochloride; Ketalar®, Pfizer, Taiwan) and xylazine (20 mg/mL; Rompun®, Bayer, Taiwan) at the left thigh. The dosage was 1 mL/Kg of the mixture. The surgical field was prepared by trimming of hair and application of 70% ethanol to the surgical site. A 1.5-cm longitudinal straight skin incision was made, then blunt dissection was carried out and fractures of 7-mm diameter were created on the skull-cap, then P1TC2 or P1TC3 was injected into the fracture sites. Intra-vital histochemical staining of the interfacial areas between the bone and bone cement were performed 4 weeks after injection, and the animals were then sacrificed at the predetermined period. All procedures employed in this study were in accordance with the standards of guidelines for the care and use of laboratory animals established and approved by the National Taiwan University College of Medicine and College of Public Health Institutional Animal Care and Use Committee (IACUC) (Approval No. 20130429).

### Statistical analyses

Results were expressed as mean ± standard deviation and statistically analyzed by two-way analysis of variance (ANOVA) with the statistical significance by the Fisher’s protected least significant difference test for multiple comparisons. The unpaired two-tailed Student’s *t* test was performed to compare differences between the groups for in vitro and ex vivo experiments. Differences were considered significant at *p* < 0.05. All analyses were performed using SPSS version 16.0 software. The statistical significance levels were set at *p* < 0.05.

## Results

**Fig. 1:**
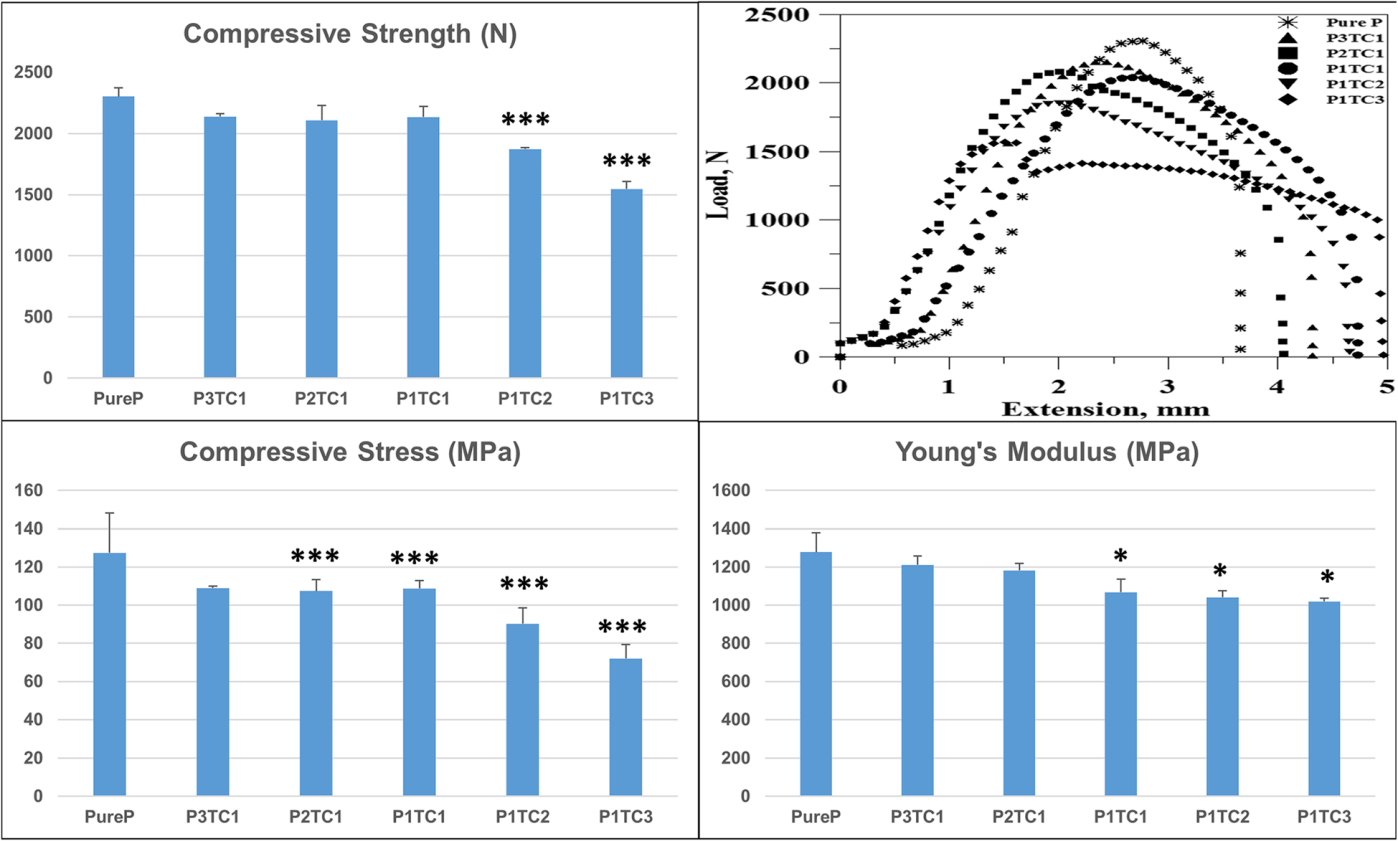
Compressive stress of bio-composite bone cements. Compressive stress of pure PMMA (Pure P) is higher than that of different bio-composite bone cements. The ultimate compressive stress of the specimens decreased from 127.4 ± 16.8 MPa for Pure P to 109.0 ± 0.6, 107.5 ± 4.9, 108.8 ± 3.5, 90.3 ± 6.7, and 72.1 ± 6.2 MPa for P3TC1, P2TC1, P1TC1, P1TC2, and P1TC3, respectively (*n* = 12, *p < 0.05, **p < 0.01, ***p < 0.001 when compared to Pure P)

**Fig. 2:**
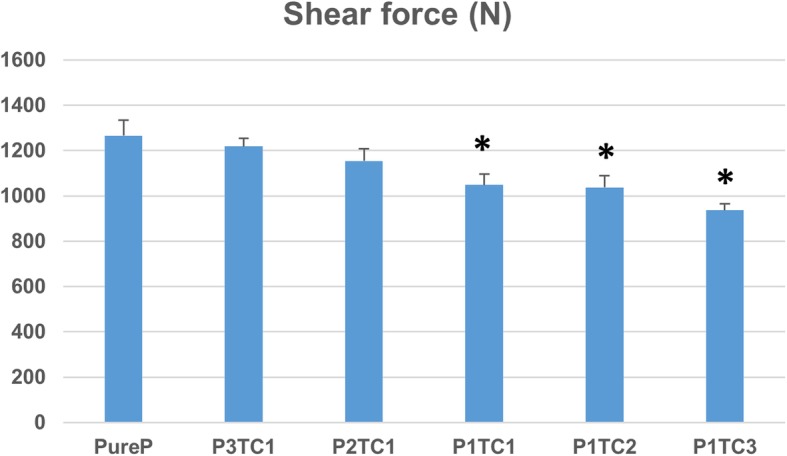
Shear force of bio-composite bone cements. The shear force between the bio-composite bone cement and metal material decreased from 1266 N for Pure P to 1048, 1037, 974, 803.6, and 797.7 N for P3TC1, P2TC1, P1TC1, P1TC2, and P1TC3, respectively (*n* = 12, *p < 0.05, **p < 0.01, ***p < 0.001 when compared to Pure P)

Compressive stress, Young’s modulus, and peak loads of pure PMMA (Pure P) are higher than that of different bio-composite bone cements. Fig. 1 revealed the ultimate compressive strength of different bio-composite bone cements. The ultimate compressive strengths of the specimens decreased from 127.4 ± 16.8 MPa for Pure P to 109.0 ± 0.6, 107.5 ± 4.9, 108.8 ± 3.5, 90.3 ± 6.7, and 72.1 ± 6.2 MPa for P3TC1, P2TC1, P1TC1, P1TC2, and P1TC3, respectively. Further, the shear force between the bio-composite bone cement and metal material also decreased (Fig. 2) from 1266 N for Pure P to 1048, 1037, 974, 803.6, and 797.7 N for P3TC1, P2TC1, P1TC1, P1TC2, and P1TC3, respectively.

**Fig. 3:**
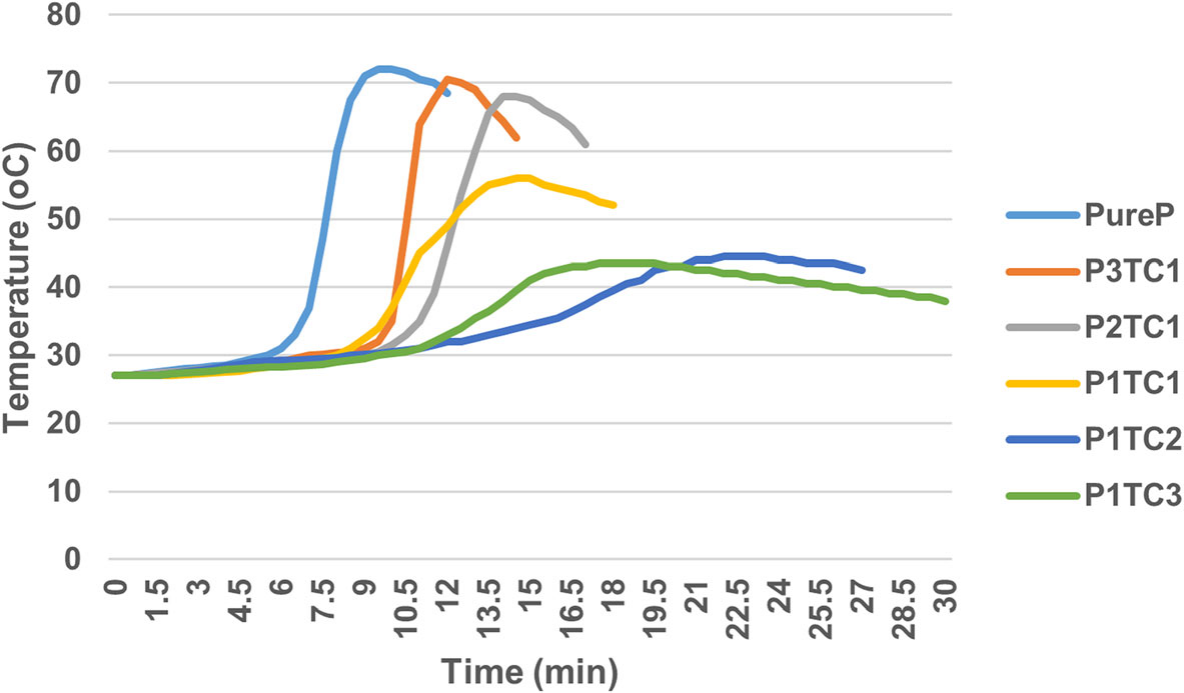
Curing temperature profiles of Pure P and bio-composite bone cements. The exothermic temperatures of the bio-composite bone cement were considerably lower than that of the pure acrylic bone cement. The maximal polymerization temperature was the highest (72.0 °C) and setting time was the shortest (7.5 min) for Pure P; while the maximal polymerization temperature was the lowest (43.5 °C) and setting time was the longest (15 min) for P1TC3

During solidification test, all the bio-composite bone cements required a relatively longer setting time to solidify when compared with acrylic bone cement (Table 2). The exothermic temperatures of the bio-composite bone cement were considerably lower than that of the acrylic bone cement (Fig. 3). The maximal polymerization temperature was the highest (72.0 °C) and setting time was the shortest (7.5 min) for Pure P; while the maximal polymerization temperature was the lowest (43.5^o^C) and setting time was the longest (15 min) for P1TC3.

**Table 2:** Mechanical properties, polymerization temperatures and the setting time of biocomposite bone cement

Item	Ultimate compressive strength (N)	Modulus of compression (MPa)	Compressive stress (MPa)	Shear force (N)	Maximum polymerization (°C)	Setting time (min)
Pure P	2302 ± 69.69	1278.3 ± 101.8	1278.3 ± 101.78	1266.3 ± 67.563	72.0	7.5
P3TC1	2140 ± 22.26	1211.4 ± 46.47	1211.6 ± 46.467	1219.1 ± 34.196	70.5	7.7
*p*-value	0.056	0.3194	0.3661	0.428		
P2TC1	2110 ± 117.5	1182.5 ± 36.10	1182.5 ± 36.096	1153.8 ± 54.932	68.0	8.2
*p*-value	0.072	0.000	0.1992	0.1416		
P1TC1	2136 ± 84.22	1142.0 ± 68.77	1067.7 ± 68.773	1048.2 ± 47.415	56.0	11.2
*p*-value	0.058	0.000	0.0411	0.0202		
P1TC2	1873 ± 14.58	1042.0 ± 33.98	1041.6 ± 33.975	1037.2 ± 52.637	44.5	13
*p*-value	0.0005	0.000	0.0188	0.0194		
P1TC3	1548 ± 61.28	1019.0 ± 18.54	1019 ± 18.544	935.99 ± 28.193	43.5	15
*p*-value	0.0001	0.000	0.0122	0.0163		

Average ± S.D., *n* = 12; *P*-value is compared to Pure P

**Fig. 4:**
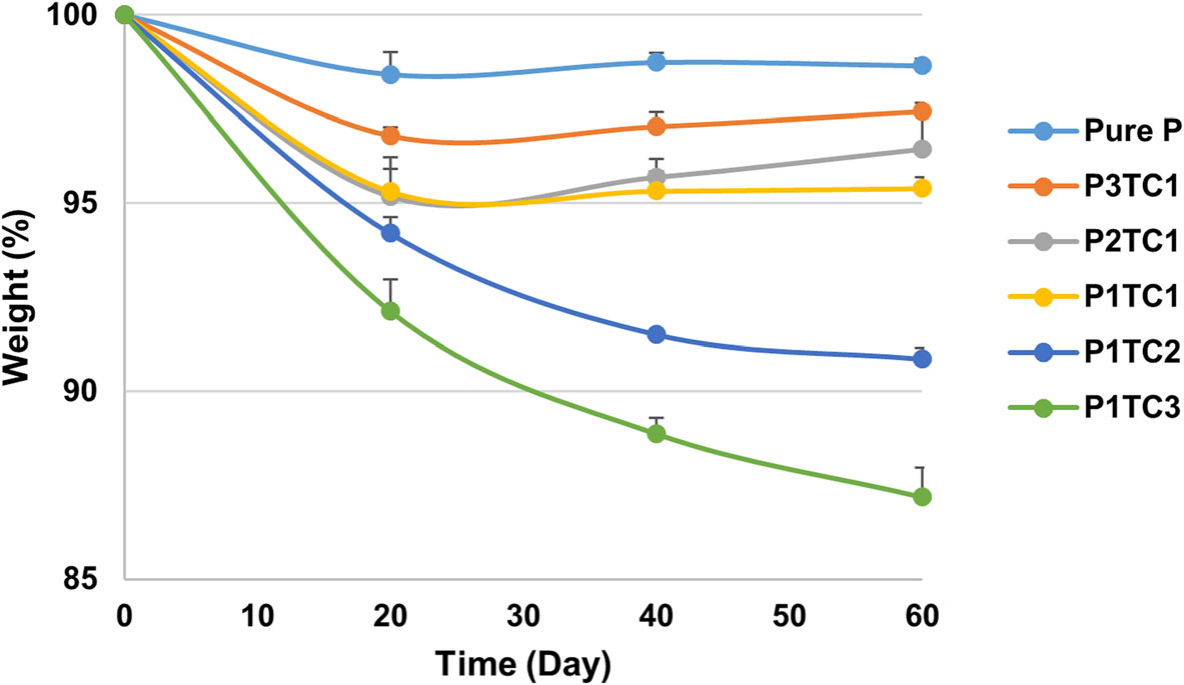
Degradation behaviors of Pure P and bio-composite bone cements. P1TC3 shows considerable weight loss (about 12.8%) compared with other bio-composite bone cements or acrylic bone cement: 2.6, 3.6, 4.6, and 9.1% for P3TC1, P2TC1, P1TC1, and P1TC2, respectively

**Fig. 5:**
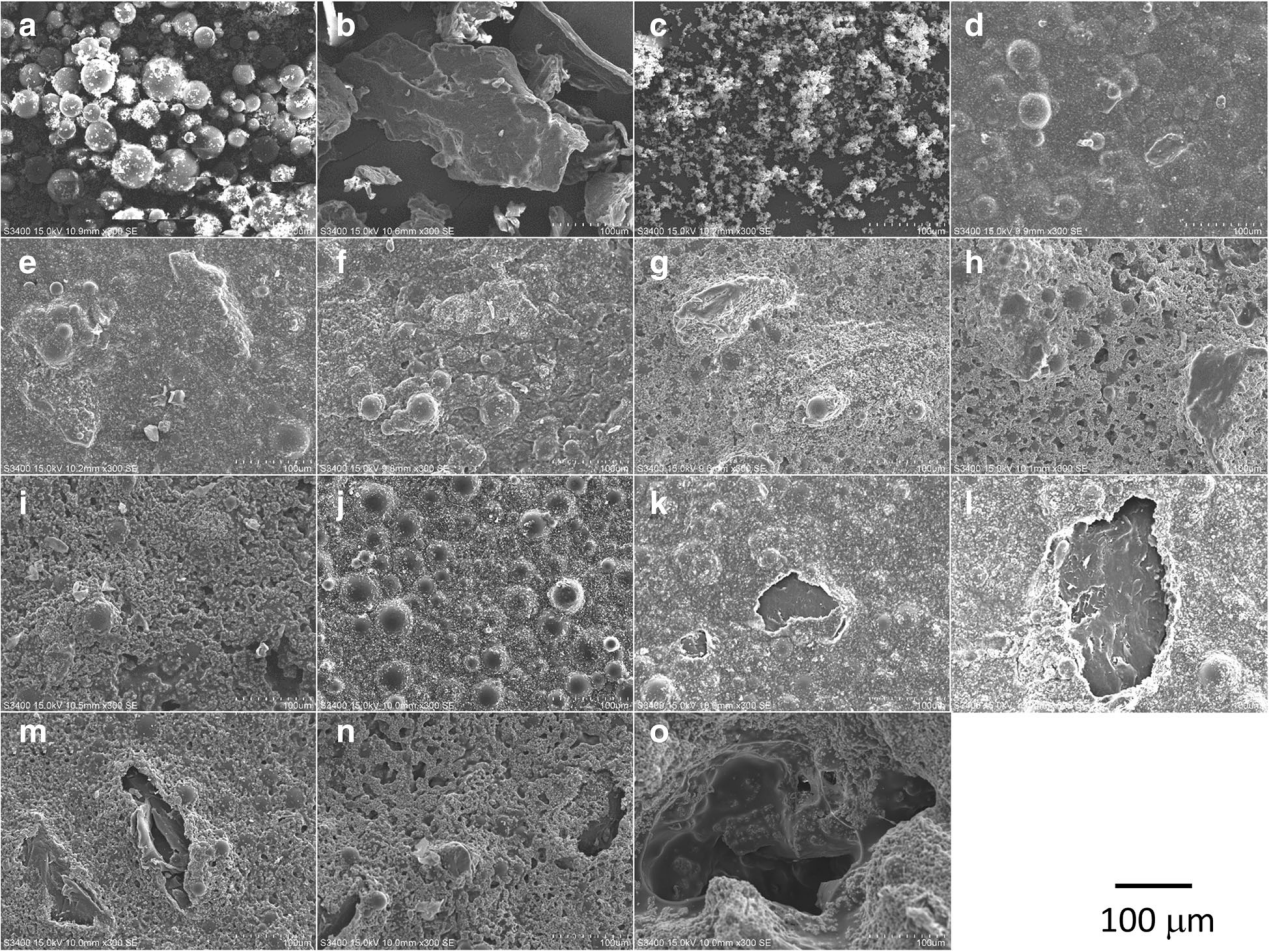
SEM images of **a** PMMA powder, **b** chitosan powder, **c** TCP powder, **d** pure P (before degradation), **e** P3TC1 (before degradation), **f** P2TC1 (before degradation), **g** P1TC1 (before degradation), **h** P1TC2 (before degradation), **i** P1TC3 (before degradation), **j** pure P (after degradation), **k** P3TC1 (after degradation), **l** P2TC1 (after degradation), **m** P1TC1 (after degradation), **n** P1TC2 (after degradation), and **o** P1TC3 (after degradation). The SEM images show that TCP and chitosan induced pores for osteo-conduction after degradation; the higher TCP and chitosan content enhanced degradation process and with larger pore size

The degradation behaviors of the prepared bio-composite bone cements are shown in Fig. 4. P1TC3 shows considerable weight loss (about 12.8%) compared with other bio-composite bone cements or acrylic bone cement: 2.6, 3.6, 4.6, and 9.1% for P3TC1, P2TC1, P1TC1, and P1TC2, respectively. The SEM images show that TCP and chitosan induced pores for osteo-conduction after degradation; the higher TCP and chitosan content enhanced more the degradation process and with larger pore size (Fig. 5).

**Fig. 6:**
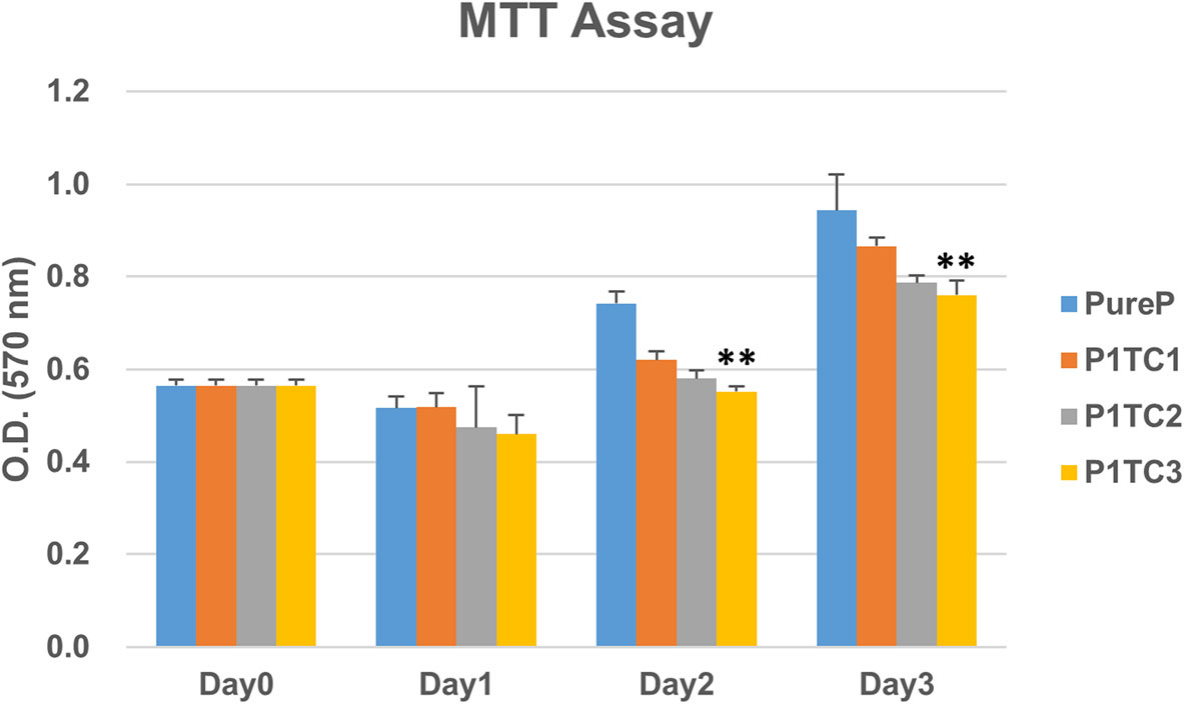
Cell viability of control and biocomposite bone cement obtained by MTT assay. The cytotoxicity results show that the cell viability of the bio-composite bone cement is not significantly different from that of the acrylic bone cement except the P1TC3 composite which showed lower cell viability (*n* = 12, *p < 0.05, **p < 0.01, ***p < 0.001 when compared to Pure P)

**Fig. 7:**
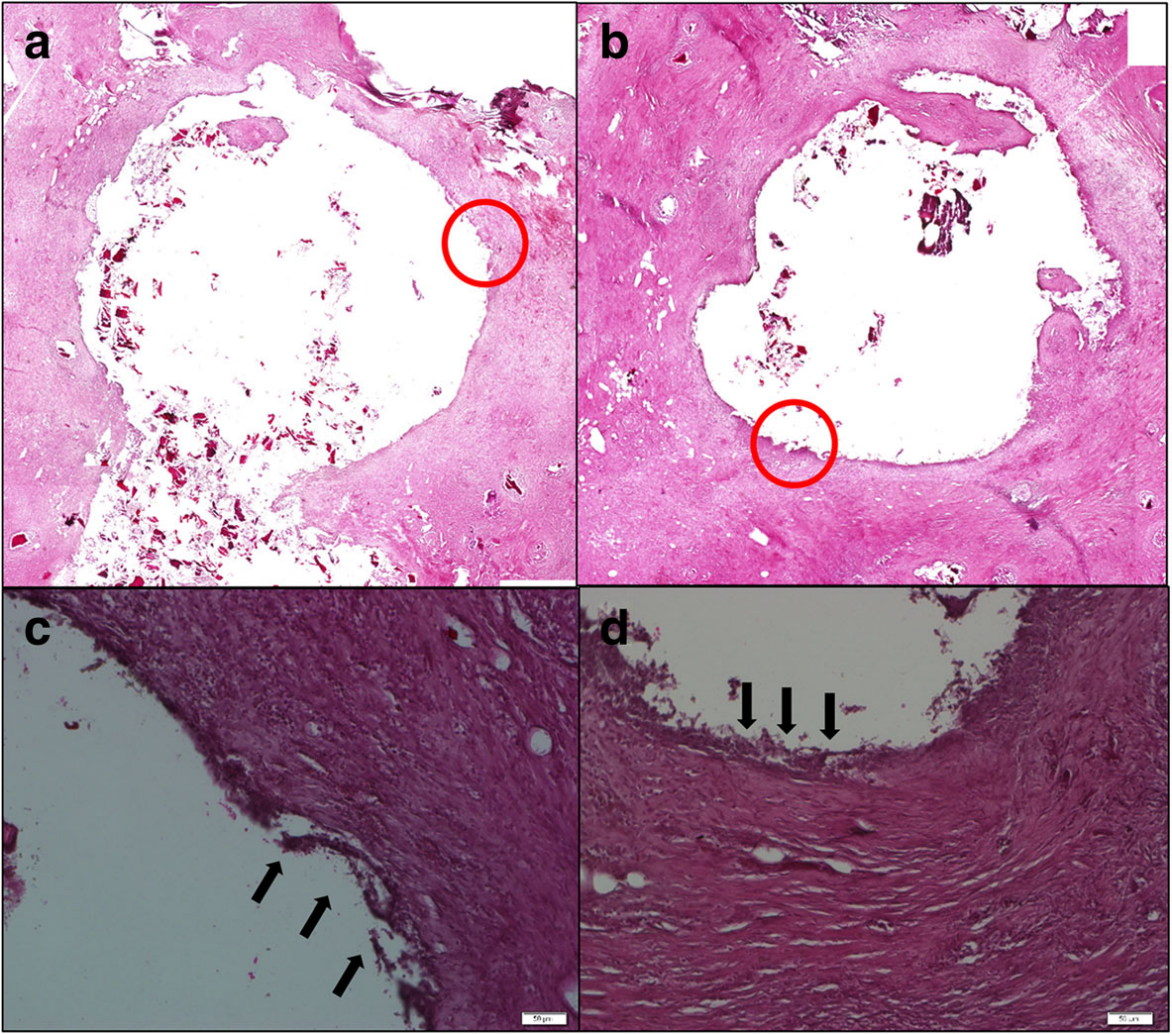
Histological examinations (H&E stain) of the interfaces between bone and bone cement after 4 weeks of implantation. **a**, **c** P1TC2 and **b**, **d** P1TC3. New bone is indicated by arrows. For the biocomposite bone cement, the in vivo histological examinations demonstrated that a large number of pores existed in the interfacial area between the host bone and the bone cement, which afforded more space for bone ingrowth

The cell viability of control and bio-composite bone cement was obtained by MTT assay. The cytotoxicity results show that the cell viability of the bio-composite bone cement is not significantly different from that of the acrylic bone cement except the P1TC3 composite which showed lower cell viability (Fig. 6). For the bio-composite bone cement, the in vivo histological examinations demonstrated that a large number of pores existed in the interfacial area between the host bone and the bone cement, which afforded more space for bone ingrowth. These resulted in that the defect size became narrower after 4 weeks of implantation, as indicated by the arrows in Fig. 7.

## Discussion

Damage to the surrounding bone tissue caused by high heat generated during its hardening polymerization process and mobility of the implant and later aseptic loosening after long-term implantation contributed by the poor compatibility with bone tissue are some of the inherent problems for the clinical use of PMMA cement in daily orthopedic practice [9]. On the other hand, bone replacement with various materials of natural origin and many different bone substitutes, such as demineralized bone matrix, platelet-rich plasma, hydroxyapatite, adjunction of growth factors (like bone morphogenetic protein) or synthetic such as calcium sulfate, tri-calcium phosphate ceramics, bioactive glasses, or polymer-based substitutes, can be used. These bone substitutes have to be chosen selectively depending on their clinical purpose. The addition of hydroxyapatite (HA) can enhance the mechanical properties of bone cement and reduce the potentially harmful heat generated during the polymerization of PMMA bone cement [23]; however, the resorption rate is much too slow in the physiological environment and thus results in an undesirable long-terminter-surfacial effect between HA ceramic and implant. From clinical practice and experience, β-TCP has a more favorable resorption pattern and osteo-transduction property than HA. Beta-TCP can be gradually absorbed and the following new bone formation is often incorporated as an additive to bone-grafting and dental materials [24].

In the attempt to improve the situation, Lin et al. fabricated degradable chitosan/beta-tri-calcium phosphate (beta-TCP) microspheres and used as an added constituent to commercially available PMMA bone cement. When compared to the PMMA cement, they demonstrated that this new composite had the beneficial characteristics of decreased the curing peak temperature and increased setting time from 3.5 to 9 min. They also found that the presence of chitosan/beta-TCP microspheres reduced the ultimate compressive strength and bending strength. The modified chitosan/beta-TCP/PMMA composites could be degraded gradually and the created rougher surfaces would be beneficial to cell adherence and growth [13]. In a similar study of bone regeneration in rabbits, by using chitosan and beta-tri-calcium phosphate(β-TCP), Azevedo et al. demonstrated that this treatment accelerated bone repair. Morphometric analysis showed that treatment groups presented statistically higher bone formation compared with the control group [25].

In this study, we proposed a bio-composite bone cement, which is composed of TCP, chitosan, and the commercial acrylic bone cement (Pure P). In this bio-composite bone cement, TCP and chitosan contents were selected to mimic the composition of human bone, i.e., 65 wt% inorganic and 35 wt% organic components. The ultimate compressive strength of Pure P is higher than that of the bio-composites (Figs. 1 and 2). The results show that the addition of TCP and chitosan powders to replace PMMA powder reduced the mechanical properties of the acrylic bone cement. These results are consistent with those of a previous study, which reported that additives reduce the mechanical properties of acrylic bone cement [13, 26]. As the structural problem called “stress-shielding” exists, the presence of two materials with significantly different mechanical properties will lead to considerable stress discontinuity, which could cause structural instability in the long term when pure acrylic bone cement was used in orthopedic surgery [11, 12]. Therefore, our results indicate that the bio-composite bone cement might experience less stress-shielding, which could be beneficial to the structural stability in the long term when compared with the pure acrylic bone cement used in orthopedic surgery.

In the clinical orthopedic practice, the too short setting time of the pure acrylic bone cement could hamper its delivery. The results of solidification tests showed that the decrease in the acrylic bone cement (PMMA) content of the bio-composite bone cements increased the setting time of the bio-composite bone cements (Table 2). Nevertheless, the average setting times for all of the bio-composite bone cement were still acceptable; moreover, the bio-composite bone cement can be more easily handled than the pure acrylic bone cement. The exothermic temperatures of the bio-composite bone cement were considerably lower than that of the pure acrylic bone cement (Fig. 3). Previous studies reported that the high polymerization temperatures of acrylic bone cement might lead to acute cell death [27], and TCP has been found to reduce the potentially harmful heat generated during the polymerization of acrylic bone cement [11, 13, 27, 28]. Therefore, our results indicate that the bio-composite bone cement with the reduced potentially harmful heat generated also might be easier to handle; these could be beneficial when used in orthopedic surgery.

The prepared biocomposite bone cements have better degradation behaviors than that of pure acrylic bone cement (Fig. 4). The presence of TCP and chitosan induced pores for osteo-conduction after degradation (Fig. 5). Porous bone cement enables cell ingrowth, enhancing compatibility with the surrounding bone tissue, which is desirable for a more stable structural anchorage of the bone cement with the surrounding tissue [29]. Previous studies have shown that the pore size for successful ingrowth of bone cells in orthopedics is at least 150 μm [30]; in this study, the bio-composite bone cement such as P1TC3 with pore sizes of about 200 μm is suitable for clinical use.

## Conclusions

The prepared bio-composite bone cements exhibited better characteristics such as low curing exothermic heat, acceptable hardening time, and good biocompatibility; retained the good mechanical properties; and, thus, also demonstrated potential for the clinical applications. In animal studies, the induced pores after degradation of TCP and chitosan may be osteo-conductive for bone cells ingrowth; this indicates that the bio-composite bone cements have potential both in osteo-conduction and osteo-integration. Our future studies will focus on long-term implantation to investigate the stability of the bio-composite bone cement after long-term implantation.

## Abbreviations

HA: Hydroxyapatite
PMMA: Polymethylmethacrylate
SEM: Scanning electron microscope (SEM)
TCP: Tri-calcium phosphate
